# Fine-Mapping Resolves Eae23 into Two QTLs and Implicates ZEB1 as a Candidate Gene Regulating Experimental Neuroinflammation in Rat

**DOI:** 10.1371/journal.pone.0012716

**Published:** 2010-09-15

**Authors:** Pernilla Stridh, Melanie Thessen Hedreul, Amennai Daniel Beyeen, Milena Z. Adzemovic, Hannes Laaksonen, Alan Gillett, Johan Öckinger, Monica Marta, Hans Lassmann, Kristina Becanovic, Maja Jagodic, Tomas Olsson

**Affiliations:** 1 Center for Molecular Medicine, Department of Clinical Neuroscience, Neuroimmunology Unit, Karolinska Institutet, Stockholm, Sweden; 2 Center for Brain Research, Medical University Vienna, Vienna, Austria; Instituto Gulbenkian de Ciência, Portugal

## Abstract

**Background:**

To elucidate mechanisms involved in multiple sclerosis (MS), we studied genetic regulation of experimental autoimmune encephalomyelitis (EAE) in rats, assuming a conservation of pathogenic pathways. In this study, we focused on *Eae23*, originally identified to regulate EAE in a (LEW.1AV1xPVG.1AV1)F2 cross. Our aim was to determine whether one or more genes within the 67 Mb region regulate EAE and to define candidate risk genes.

**Methodology/Principal Findings:**

We used high resolution quantitative trait loci (QTL) analysis in the 10th generation (G10) of an advanced intercross line (AIL) to resolve *Eae23* into two QTLs that independently regulate EAE, namely *Eae23a* and *Eae23b*. We established a congenic strain to validate the effect of this region on disease. PVG alleles in *Eae23* resulted in significant protection from EAE and attenuated CNS inflammation/demyelination. Disease amelioration was accompanied with increased levels of Foxp3^+^ cells in the CNS of the congenic strain compared to DA. We then focused on candidate gene investigation in *Eae23b*, a 9 Mb region linked to all clinical phenotypes. Affymetrix exon arrays were used to study expression of the genes in *Eae23b* in the parental strains, where none showed differential expression. However, we found lower expression of exon 4 of *ZEB1,* which is specific for splice-variant *Zfhep1*. *ZEB1* is an interleukin 2 (*IL2*) repressor involved in T cell development. The splice-specific variance prompted us to next analyze the expression of *ZEB1* and its two splice variants, *Zfhep1* and *Zfhep2*, in both lymph node and spleen. We demonstrated that *ZEB1* splice-variants are differentially expressed; severity of EAE and higher *IL2* levels were associated with down-regulation of *Zfhep1* and up-regulation of *Zfhep2.*

**Conclusions/Significance:**

We speculate that the balance between splice-variants of *ZEB1* could influence the regulation of EAE. Further functional studies of *ZEB1* and the splice-variants may unravel novel pathways contributing to MS pathogenesis and inflammation in general.

## Introduction

Multiple Sclerosis (MS) is a chronic inflammatory disease of the central nervous system (CNS), classified as a complex immunopathological disease that depends on interactions between genetic and environmental factors [1], [2]. Observations in twin cohorts and familial aggregation studies have demonstrated the genetic component of MS etiology [3]–[5] and an influence from genes within the major histocompatibility complex (MHC, HLA in humans) is well established [6]–[10]. Recently, a few non-HLA genes have unambiguously been associated with MS susceptibility, including *IL2RA* (ENSG00000134460), *IL7R* (ENSG00000168685), *CD58* (ENSG00000116815) and *CLEC16A* (ENSG00000038532) [11]–[15]. The ongoing challenge is to elucidate the molecular mechanisms conferred by the risk genes. Therefore, analyses in a simplified system are advocated.

The genetic dissection of a rat model for MS represents such an approach, with the assumption that there are conserved mechanisms regulating neuroinflammation among species. We study the genetic regulation of myelin oligodendrocyte glycoprotein (MOG) induced experimental autoimmune encephalomyelitis (EAE) in rats due to a close mimicry with MS, with a protracted relapsing disease course, inflammatory infiltrates, prominent demyelination and axonal damage [16]. Consistent with MS, the MHC locus is suggested to be the strongest susceptibility locus in EAE [17]. Furthermore, non-MHC loci that contribute to EAE susceptibility have been identified in several F2 crosses using different strain combinations [18]–[20]. However, the determination of disease-regulating genes requires further fine-mapping and validation approaches.

Here we use an approach combining high-resolution mapping in an advanced intercross line (AIL) with congenic validation and gene expression studies. This approach has successfully been used to identify EAE-regulating genes. An influence of *Eae18* on MOG-EAE was confirmed in a congenic strain [21] and the region was fine-mapped in the AIL to identify a cluster of chemokine genes as candidate genes [22]. Further investigation demonstrated association of *CCL1* (ENSG00000108702), *CCL2* (ENSG00000108691) and *CCL12* (ENSRNOG00000029768) with MS [23]. Here we focused on another region, on rat chromosome 17 (RNO17) that was originally identified in a (LEW.1AV1xPVG.1AV1)F2 cross, where linkage analysis revealed a quantitative trait locus (QTL) regulating clinical and histopathological signs of EAE (named *Eae23* in this study) [20].

Our aim was to determine whether one or more genes within the 67 Mb region of *Eae23* regulate EAE and to fine-map those genes. *Eae23* was resolved into two QTLs that independently regulate EAE, namely *Eae23a* and *Eae23b*. The effect of *Eae23* was confirmed in a congenic strain, DA.PVG-*Eae23*, and histopathological investigation of the CNS showed that inflammation and demyelination was attenuated compared to DA. We identified *ZEB1* (*Zfhep, TCF8, δEF1, ZFHX1A,* ENSRNOG00000017863) as the most likely candidate gene in *Eae23b*. *ZEB1* is foremost known as a repressor of interleukin 2 (*IL2,* ENSRNOG00000017348), a cytokine involved in proliferation, apoptosis, cell differentiation and T cell development at multiple stages [24], [25]. We further postulate that the balance between splice variants of *ZEB1* could influence the regulation of EAE.

## Results

### Determination of *Eae23a* and *Eae23b* using an Advanced Intercross Line

**Figure 1:**
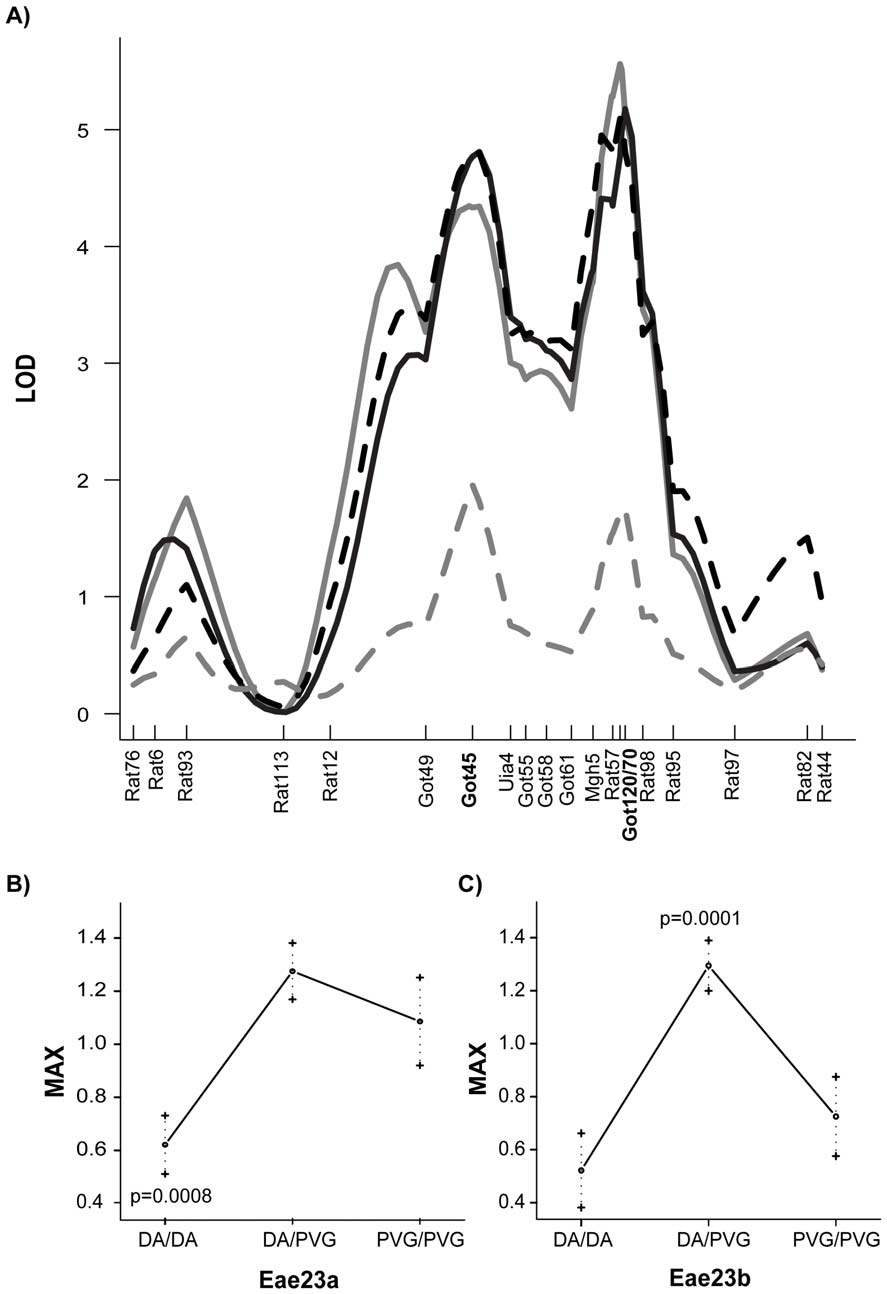
Two QTLs regulate EAE: *Eae23a* and *Eae23b*. A) A log-likelihood plot of *Eae23* regulating selected EAE phenotypes in (DAxPVG.1AV1)G10 AIL females (N = 428). Peak markers are D17Got45 for *Eae23a* and D17Got120 (ONS and DUR) and D17Got70 (INC, MAX and CUM) for *Eae23b*, and both QTLs displayed linkage to all clinical phenotypes. Microsatellite marker positions are indicated by vertical lines on the x-axis, retrieved from Ensembl Genome Database (http://www.ensembl.org v.55). B–C) Allelic effect plots for *Eae23a* and *Eae23b* in females (N = 428) show rats stratified according to their genotype at D17Got45 and D17Got70. MAX is representative for all clinical phenotypes. Phenotype codes: ONS  =  grey, MAX  =  black, DUR  =  black dashed, WL  =  grey dashed.

To narrow the confidence interval of *Eae23* [20], and confirm the locus in a new strain combination, we investigated the region in the (DAxPVG.1AV1)G10 AIL [26]–[29]. In total, 224 out of 772 AIL animals displayed clinical EAE, with an incidence of 29% (Figure S1). G10 AIL rats were genotyped over 67.7 Mb of RNO17 (13.9–81.6 Mb) using 20 polymorphic microsatellite markers (Table S1). Linkage analysis was performed in females due to a lack of power to detect the QTL in males (Figure S2). Analysis of clinical phenotypes identified two QTLs, denominated *Eae23a* and *Eae23b* (Figure 1).

*Eae23a* was linked to all clinical phenotypes and constituted a 21 Mb region around peak marker D17Got45 at 47.3 Mb (Table 1). The confidence interval was likely overestimated owing in part to all 11 microsatellite markers tested between D17Rat12 (33.3 Mb) and D17Got49 (42.7 Mb) being non-polymorphic in the strains used. The allelic effect showed transgressive segregation with alleles from the resistant PVG strain promoting disease in a dominant manner (Figure 1B). Rats homozygous for DA alleles were protected against EAE with lower incidence (p<0.0002), later onset (p<0.0005), shorter duration of disease (p<0.0004) and with lower maximum and cumulative EAE scores (p<0.0008 and p<0.0006, respectively) when compared to rats with heterozygote or PVG homozygote genotype in *Eae23a*.

**Table 1:** Linkage of *Eae23*.

Phenotype	*Eae23a* LOD Score	Peak Marker *Eae23a* a	LOD S.I. = 1.5^b^	*Eae23* Prob^c^	*Eae23b* LOD Score	Peak Marker *Eae23b* a	LOD S.I. = 1.5^b^	*Eae23* Prob^c^
INC (1.9)	3.7	D17Got45*	36–57	22.4%	4.9	D17Got70**	59–66	69.2%
ONS (2.2)	4.4	D17Got45*	36–56	27.1%	5.6	D17Got120**	59–66	66.5%
MAX (2.4)	4.8	D17Got45**	36–56	37.1%	5.2	D17Got70**	57–64	55.5%
DUR (2.0)	4.8	D17Got45**	39–51	27.7%	5.1	D17Got120**	57–64	58.7%
CUM (1.7)	4.3	D17Got45*	43–54	31%	4.2	D17Got70*	57–64	51%

Linkage of *Eae23* in 428 (DA × PVG.1AV1)G10 AIL female rats. The significance thresholds are indicated in parenthesis and are the maximum LOD scores obtained from the analysis of residual values from family mean data for each phenotype.

a Peak marker indicates the microsatellite marker closest to the highest LOD score obtained within the QTL. The fit multiple QTL model was used to validate the independent effects of *Eae23a* and *Eae23b* (phenotype  =  *Eae23a + Eae23b + ε*) and the locations used corresponds to the peak markers. A significant difference in the phenotypic variance defines an independent QTL, and is indicated by

* = p<0.05 and

** = p<0.01.

b A drop in LOD score of 1.5 from the maximum LOD (LOD support interval) was used to define the confidence intervals. Positions are given in Mb.

c Probabilities were calculated from simulated pedigrees as the percentage of times the maximum LOD was located within the confidence interval of each QTL. Abbreviations: INC  =  incidence of EAE, ONS  =  onset of EAE, MAX  =  maximum EAE score, DUR  =  duration of EAE, CUM  =  cumulative EAE score, S.I  =  Support Interval.

*Eae23b* was also linked to all clinical phenotypes, with a confidence interval of 9 Mb (57–66 Mb) around peak markers D17Got120 and D17Got70 at 61.8–62.3 Mb (Table 1). Analysis of allelic effects revealed significant heterosis whereby heterozygous alleles differ from either parental allele (Figure 1C). Rats heterozygous in *Eae23b* were predisposed to EAE with higher incidence (p<0.0001), earlier onset (p<0.0001), longer duration (p<0.0001) and more severe disease (MAX p<0.0001, CUM p<0.0001) when compared to rats with homozygous DA or PVG genotype in *Eae23b*. A striking 43% of *Eae23b*-heterozygous rats were affected by EAE, compared to only 27% of the homozygous rats.

The individual effects of *Eae23a* and *Eae23b* on EAE phenotype variation were further supported by a fit multiple QTL model [30] (Table 1). To estimate the probability of these QTLs contributing to EAE, simulated pedigrees were mapped using a single-QTL model (to force the QTLs to compete for effect). Consistent with the linkage analysis, *Eae23a* and *Eae23b* were both probable to influence susceptibility and severity phenotypes (Table 1).

### Confirmation of *Eae23* Effects on EAE using Congenic Rats

**Figure 2:**
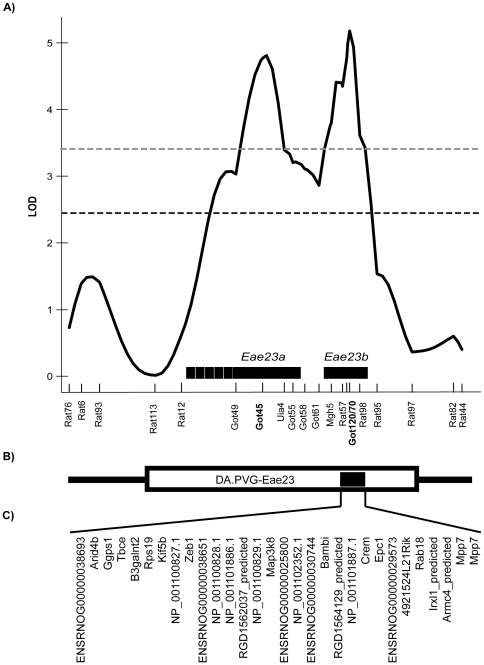
Summary of the *Eae23* region on RNO17. A) Summary log-likelihood plot of *Eae23*. The black dashed line depicts the significance threshold determined by the analysis of residual family values and the grey dashed line depicts a threshold of 3.4 [30]. Horizontal bars above the *x-*axis indicate the confidence intervals of each QTL according to a 1.5 LOD support interval (clinical phenotypes are represented by MAX). Microsatellite marker positions are indicated by vertical lines on the x-axis, retrieved from Ensembl Genome Database (http://www.ensembl.org v.55). B) The open box below the *x*-axis represents the region where PVG alleles were introgressed into a DA background in the DA.PVG-*Eae23* congenic strain tested in MOG-EAE. The black box indicates the confidence interval of *Eae23b*. C) The candidate genes investigated in this study: 30 genes contained within *Eae23b.*

**Figure 3:**
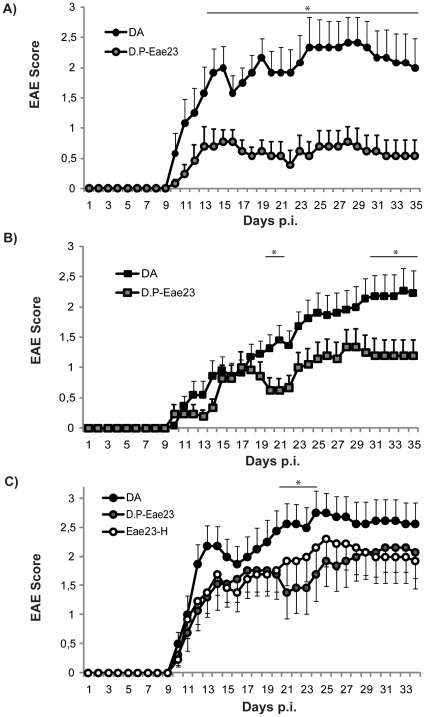
A congenic strain confirms the influence of *Eae23* on EAE in females and males. Daily EAE scores in parental DA and congenic DA.PVG-*Eae23* demonstrate that congenic strains with PVG alleles in *Eae23* are protected from EAE. A) Females; DA N = 12 and DA.PVG-*Eae23* N = 13. B) Males; DA N = 22 and DA.PVG-Eae23 N = 21. C) Females; DA N = 16, DA.PVG-*Eae23* N = 13 and heterozygous DA.PVG-Eae23 N = 13. Females are represented by circles and males are represented by squares. Mann-Whitney U-test was used to compare the daily EAE scores; *p* values ≤0.05 were considered significant and is indicated by *. Abbreviations: D.P-Eae23 =  DA.PVG-*Eae23*, Eae23-H  =  heterozygous DA.PVG-*Eae23*, p.i.  =  post immunization. Vertical bars represent standard error of the mean (SEM).

To confirm the influence of *Eae23* on disease we established DA.PVG-*Eae23*, a congenic strain that carries PVG alleles in *Eae23a* and *Eae23b* introgressed on a DA background (Figure 2). Commonly, the parental DA strain displays a relapsing-remitting disease course with an average onset of EAE around two weeks after immunization, while the parental PVG.1AV1 strain is resistant to the same induction protocol. DA.PVG-*Eae23* littermate controls showed no clinical differences to inbred DA (data not shown) and DA were therefore used as control in the experiments. To follow up the AIL findings we first investigated the clinical effects in females during MOG-EAE. In total, 20 of the 25 female rats included in the experiment showed signs of EAE (80% incidence). Although there was no significant difference in susceptibility (INC and ONS) between strains, DA.PVG-*Eae23* rats had significantly less severe clinical symptoms compared to DA during both the acute and chronic phase of disease (Figure 3A). DA.PVG-*Eae23* female rats had less severe disease, with lower maximum (p<0.02) and cumulative (p<0.007) EAE scores and shorter duration of disease (p<0.01) (Table 2). In total, 34 of 43 male rats included in the experiment developed EAE (79% incidence) which was comparable with the incidence in females. DA.PVG-*Eae23* males had less severe disease during the acute and chronic phases compared to DA, and had a lower maximum EAE score (p<0.04) (Figure 3B, Table 2).

**Table 2:** DA.PVG-*Eae23* is protected from EAE compared to DA.

	Females Mean (SD)		Males Mean (SD)	
	DA (N = 12)	D.P-*Eae23* (N = 13)	DA (N = 22)	D.P-*Eae23* (N = 21)
Incidence	92%	69%	82%	76%
Day of Onset	14.8 (8.3)	22.5 (12.9)	19.6 (10.4)	21.9 (11.3)
Max EAE Score	2.8 (1.4)	1.5 (1.3)*	2.6 (1.6)	1.7 (1.2)*
Duration	19.7 (8.6)	10.1 (9.5)*	15.8 (8.9)	11.7 (10.2)
Cumulative EAE score	50.7 (32.7)	15.0 (17.0)**	38 (28)	23 (23.9)

Mann-Whitney U-test was used to compare clinical phenotypes between parental DA and DA.PVG-*Eae23*; *p* values ≤0.05 were considered significant, indicated by

* = p<0.05;

** = p<0.01. Abbreviations: SD  =  standard deviation.

To investigate if the heterosis effect of *Eae23b* detected in the AIL could be captured in the congenic strain, we repeated the experiment with heterozygous DA.PVG-*Eae23* rats. Based on the AIL results we expected heterozygous DA.PVG-*Eae23* rats to have the full effect of both *Eae23a* and *Eae23b* (PVG dominance and heterosis, respectively) with a more severe disease, while homozygous rats should show effect of *Eae23a* only (PVG dominance) and have an intermediate phenotype. However, there were no significant differences between heterozygous DA.PVG-*Eae23* and DA (Figure 3C). Although the homozygous DA.PVG-*Eae23* rats were significantly protected compared to DA (consistent with the previous experiments), more rats in total were affected by EAE (93% incidence) and the congenic rats showed slightly increased disease severity. Since both female experiments were immunized with the same protocol, this slight variation may reflect inter-experiment fluctuation or a seasonal influence (winter compared to spring) on EAE [31]. To understand the lack of effect in heterozygous congenic rats, we selected individuals from the AIL cohort that matched the genotype combinations captured in the congenic rats (Table 3). This analysis was consistent with the results from congenic rats, since the allele combination captured in the heterozygous congenic rats also produced an intermediate phenotype in the AIL that was not significantly different from the phenotypes of rats with DA genotype. We were therefore unable to further investigate the heterosis effect in the congenic strain.

**Table 3:** Expected congenic phenotype values based on the (DA × PVG.1AV1) G10 AIL.

Congenic Strain	AIL Genotype		Expected Phenotype Value					
	*Eae23a*	*Eae23b*	WL0^b^	INC a	ONS b	MAX b	DUR b	CUM b
DA (62)	DA	DA	0.95	17%	35.88	0.36	1.67	3.59
D.P-*Eae23* (98)	PVG	PVG	3.45**	47%***	27.59***	1.35***	7.93***	19.36***
*Eae23*-Het (30)	DA/PVG	DA/PVG	2.57	35%*	30.61	1.03	5.9	14.29

Rats that matched the genotype combinations captured in the congenic strains were selected from the AIL cohort. The number of AIL rats that were used to generate the expected phenotype value for each congenic strain is indicated in parenthesis. Rats with DA genotype were compared to rats with either PVG or heterozygous genotype in *Eae23*;

a =  Fisher's Exact Test,

b =  Mann-Whitney U-test. *p* values ≤0.05 were considered significant, indicated by

* = p<0.05;

** = p<0.01;

*** = p<0.001.

### Influence of *Eae23* on CNS pathology in Congenic Rats

**Figure 4:**
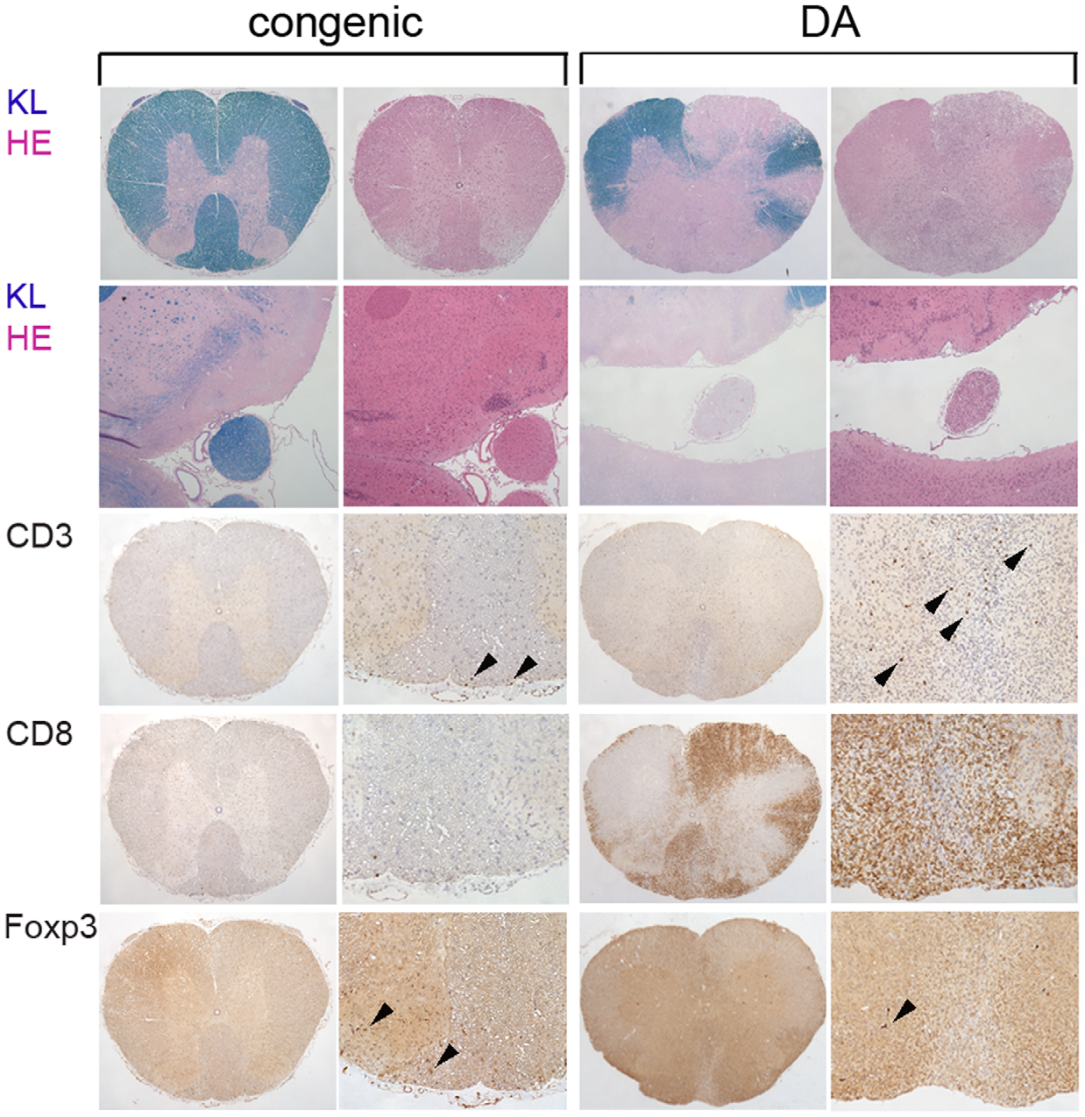
CNS histopathology demonstrates less inflammation and demyelination in DA.PVG-*Eae23* than DA. Spinal cord and brain sections of DA.PVG-*Eae23* congenic rats (the two left columns) and DA rats (the two right columns) with the same MAX score. Sections were stained in the following order: spinal cord cross-sections (top row) and brain sections including optic nerve (row 2) were stained with Klüver (KL) and Hämalaun*-*Eosin (HE), respectively. Congenic rats revealed no signs of CNS inflammation and demyelination while DA rats showed severe loss of myelin and cell infiltration at the sight of the lesion in the spinal cord white matter, as well as at the optic nerve. Staining of the spinal cord sections against CD3 (row 3), CD8 (row 4) and Foxp3 (bottom row) revealed the higher recruitment of CD3^+^ T cells and CD8^+^ macrophages respectively to inflammatory lesions in the DA rats and higher recruitment of Foxp3^+^ T cells in DA.PVG-*Eae23* congenic rats. Arrow heads point to cells with positive staining and indicate the relative number of positive cells present.

To evaluate the effects of *Eae23* on EAE pathology in the target organ, we investigated inflammation and demyelination in the CNS of DA and DA.PVG-*Eae23* female rats at 35 days post immunization (p.i.). Immunohistopathological analysis of the brain and the spinal cord revealed differences in inflammation/demyelination, as well as in cell activation and infiltration between strains (Figure 4). The most frequent distribution pattern (41% overall) was characterized by the combined appearance of demyelinated lesions in the optic nerves and the spinal cord. The control group (DA) developed more severe disease compared to the congenic strain (Table 4). However, when we compared rats with the same MAX and ONS, congenic rats showed generally lower susceptibility for CNS inflammation with a lower degree of demyelination and with a lower number of eosinophils and infiltrating CD8^+^ macrophages [32]. We also observed a lower recruitment of the CD3^+^ T cell population to the spinal cord in congenic rats (indicated by arrow heads in Figure 4). The distribution pattern of CD3^+^ T cells, CD8^+^ macrophages and eosinophils corresponded to the demyelinated lesions in the brain and in the spinal cord. There was significantly less demyelination in both brain and spinal cord of DA.PVG-*Eae23* rats compared to DA (p<0.01 and p<0.03, respectively). Interestingly, Foxp3^+^ T cells were present in higher numbers in the congenic rats compared to DA (indicated by arrow heads in Figure 4).

**Table 4:** CNS Histopathology demonstrates protection from EAE in DA.PVG-*Eae23*.

	DA	DA.PVG-*Eae23*
	Mean (SD)	Mean (SD)
Weight at immunization (grams)	162.9 (9.5)	153.5 (16.7)
Adjusted dose (µg MOG/g rat)	0.092 (0.005)	0.099 (0.011)
Incidence (%)	89 (33)	69 (48)
Day of Onset (day p.i.)	15.6 (9.4)	22.5 (12.9)
Max EAE Score	2.7 (1.2)	1.5 (1.3)*
Duration (days)	20 (8.5)	10.1 (9.5)*
Cumulative EAE score	46.8 (28.4)	15 (17)*
Spinal Cord Demyelination	1.8 (1.6)	0.09 (0.3)*
Spinal Cord Inflammation Index	0.58 (0.46)	0.14 (0.29)*
Brain Demyelination	2.6 (1.8)	0.35 (1.1)*
Demyelination in Optic Nerves	7/9	2/13*
Pons Involvement	1/9	0/13

Mann-Whitney U-test was used to compare histopathology phenotypes between parental DA and DA.PVG-*Eae23*; *p* values ≤0.05 were considered significant, indicated by *.

Abbreviations: SD  =  standard deviation, p.i.  =  post immunization.

### Influence of *Eae23* on regulatory T cell response to MOG recall

**Figure 5:**
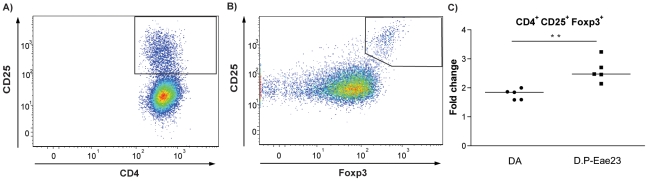
Regulatory T cell response to MOG recall in early disease. Day 7 p.i. lymph node cells from DA and DA.PVG-*Eae23* were stained for T reg markers and pre-gated for CD4^+^ T cells using flow cytometry. A) Representative flow cytometry plot of CD4 (x-axis) and CD25 (y-axis) showing the gate for the CD4^+^CD25^+^ population. B) Representative flow cytometry plot of Foxp3 (x-axis) and CD25 (y-axis) showing the gate for the CD4^+^CD25^+^Foxp3^+^ population. C) Fold change of CD4^+^CD25^+^Foxp3^+^ sub-populations after MOG recall compared to ex vivo. Median values are indicated by horizontal bars. Mann-Whitney U-test was used to compare the fold change; *p* values ≤0.05 were considered significant, **  = p≤0.01. Abbreviations: D.P-Eae23 =  DA.PVG-*Eae23.*

To investigate the possibility of an enhanced population of Foxp3^+^ regulatory T cells (Tregs) in DA.PVG-*Eae23*, we determined the proportion of Foxp3-expressing T cells in lymph nodes collected 7 days p.i. (Figure 5). Using flow cytometry, a higher proportion of CD4^+^CD25^+^Foxp3^+^ cells was detected in ex vivo lymph node cells from the susceptible DA strain compared to DA.PVG-*Eae23* (N = 5 per group; p = 0.008; data not shown). However, in response to MOG restimulation, DA.PVG-*Eae23* had a significantly higher up-regulation of the CD4^+^CD25^+^Foxp3^+^ population than DA (2.6- and 1.8-fold change, respectively; p = 0.008; Figure 5C).

### Expression Studies Identify *ZEB1* as a Candidate EAE-regulating gene

**Figure 6:**
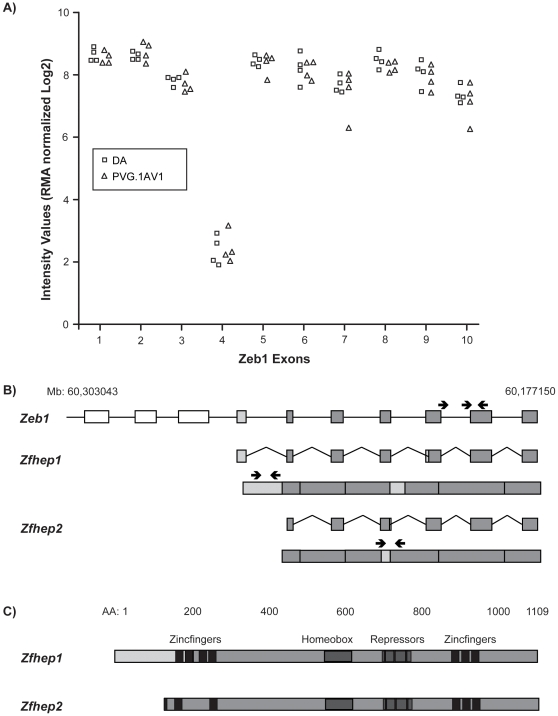
Exon-level mRNA expression of ZEB1 in DA and PVG.1AV1 lymph nodes 7 days p.i. A) Exon-level expression of *ZEB1* demonstrate that exon 4 had lower expression compared to all other exons. B) Schematic illustration of the splice variants *Zfhep1* and *Zfhep2* of rat *ZEB1* based on mRNA sequence. Exon 4 is specific for the *Zfhep1* variant. White boxes represent untranslated exons, light grey boxes represent splice variant-specific exons or sequences and dark grey boxes represent shared exons. C) Schematic illustration of the protein structure of *Zfhep1* and *Zfhep2* based on amino acid sequence. Zinc finger 1 and part of zinc finger 2 are spliced out of *Zfhep2.*

*Eae23a* contained 140 genes while *Eae23b* presented a limited number of candidate genes to investigate further. On the assumption that a shared gene is responsible for linkage between *Eae23b* and all clinical phenotypes, we restricted the candidate gene list to the region shared among clinical phenotypes. The 5 Mb region (59–64 Mb) contained 30 genes, of which 21 had described functions (Figure 2). mRNA expression of all candidate genes was investigated in lymph nodes collected day 7 p.i. from parental DA and PVG.1AV1 rats (N = 4 per group) using exon arrays [33]. There were no differences in gene-level expression of the candidate genes between strains that reached genome-wide significance. When we investigated exon-level expression of the genes, there were no differences between parental strains. However, exon 4 of *ZEB1* had much lower expression compared to all other exons of this gene, indicating alternative splicing (Figure 6A). Interestingly, *ZEB1* produces two splice variants, *Zfhep1* (*NP_037296.1,* ENSRNOT00000046013) and *Zfhep2* (*ZEB1,* ENSRNOT00000024336), for which exon 4 (*NP_037296.1* exon 1) is specific for the *Zfhep1* variant (Figure 6B). The resulting protein differs in the first zinc finger cluster (Figure 6C). This prompted us to investigate if splice-variant specific differences of *ZEB1* could be involved in regulation of EAE.

**Figure 7:**
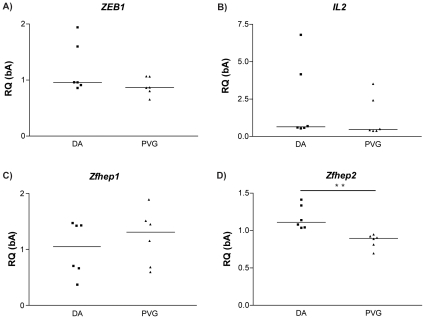
Gene expression of candidate pathway genes in parental DA and PVG.1AV1. mRNA expression in lymph nodes collected day 7 p.i. from parental DA and PVG.1AV1 reveal differential gene expression of *ZEB1* isoform *Zfhep2*. Median values are indicated by horizontal bars. Mann-Whitney U-test was used to compare the mRNA expression; *p* values ≤0.05 were considered significant, **  = p≤0.01. Abbreviations: RQ  =  relative quantity. Gene names: *ZEB1*  =  Zinc finger E-box-binding homeobox 1, *IL2*  =  Interleukin 2, *Zfhep1*  =  Zinc finger homeodomain enhancer-binding protein 1 (*NP_037296.1,* splice variant 1) and *Zfhep2*  =  Zinc finger homeodomain enhancer-binding protein 2 (*ZEB1,* splice variant 2).

To explore potential differences in *ZEB1* using the more sensitive quantitative real-time polymerase chain reaction (RT-PCR) method, we analyzed the mRNA expression of *ZEB1* and its target *IL2* in lymph nodes collected at day 7 p.i. from MOG-immunized DA and PVG.1AV1 rats (N = 6 per group). There were no significant differences between the strains in either the shared region of *ZEB1* or its downstream target *IL2* (Figure 7). However, mRNA expression of the splice variant *Zfhep2* was higher in DA compared to PVG.1AV1 (p<0.005) while there was no significant difference in *Zfhep1*.

**Figure 8:**
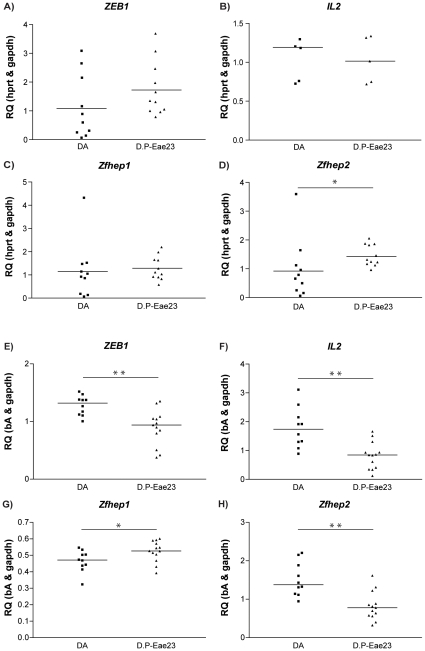
Differential *ZEB1* isoform expression in DA.PVG-*Eae23* and DA. Differential gene expression of *ZEB1* is driven by a splice-variant specific shift in balance. In lymph nodes collected day 7 p.i. from parental DA and DA.PVG-*Eae23* (A – D), mRNA expression of *Zfhep2* is up-regulated in the congenic strain compared to parental DA. In spleen collected day 35 p.i. (E – H), *Zfhep1* is up-regulated while *Zfhep2*, *ZEB1* and *IL2* are down-regulated in DA.PVG-*Eae23* compared to parental DA. Median values are indicated by horizontal bars. Mann-Whitney U-test was used to compare the mRNA expression; *p* values ≤0.05 were considered significant, *  = p≤0.05 and **  = p≤0.01. Abbreviations: D.P-Eae23 =  DA.PVG-*Eae23,* RQ  =  relative quantity. A and E) *ZEB1*  =  Zinc finger E-box-binding homeobox 1, B and F) *IL2*  =  Interleukin 2, C and G) *Zfhep1*  =  splice variant 1 of *ZEB1* (*NP_037296.1*) D and H) *Zfhep2*  =  splice variant 2 of *ZEB1* (*ZEB1*).

To investigate if *Eae23* regulates this expression we then measured *ZEB1*, splice variants and *IL2* mRNA expression in lymph nodes collected at day 7 p.i. from MOG-immunized DA and DA.PVG-*Eae23* female rats (N = 10 and N = 11, respectively) (Figure 8A–D).

No significant differences between the strains were seen in *ZEB1, Zfhep1* or *IL2*. However, mRNA expression of the splice variant *Zfhep2* was higher in DA.PVG-*Eae23* (p<0.018) compared to DA (Figure 8D), confirming regulation by the *Eae23* locus. Similarly, we observed a trend of lower secreted IL-2 in DA.PVG-*Eae23* upon recall, although differences were not significant (Figure S3).

To further investigate the expressional differences in another tissue at a later timepoint, we collected spleens from DA and DA.PVG-*Eae23* female rats on day 35 p.i (N = 9 and N = 13) (Figure 8E–H). The shared region of *ZEB1, Zfhep2* and *IL2* were significantly up-regulated in DA compared to DA.PVG-*Eae23* (p<0.001, p<0.002 and p<0.002, respectively), while *Zfhep1* was significantly down-regulated in DA compared to DA.PVG-Eae23 (p<0.04).

Our data suggests that the balance in expression between *Zfhep1* and *Zfhep2* could contribute to disease protection in the congenic strain, both in early initiation and ongoing disease.

## Discussion

In this study, we used the AIL to resolve *Eae23* into two narrow QTLs that independently regulate EAE, namely *Eae23a* and *Eae23b*. An influence of *Eae23* on neuroinflammation was then confirmed with a congenic strain. These approaches complemented each other to provide evidence for *Eae23*‘s importance for neuroinflammation.

The congenic strain carrying PVG alleles in *Eae23* demonstrated protection from clinical symptoms of disease and attenuated CNS inflammation and demyelination. This was somewhat surprising, as DA alleles in *Eae23* were disease protective in the AIL. Further, the combination of *Eae23a* DA/PVG alleles together with *Eae23b* DA/PVG alleles (disease-driving in the AIL) produced an intermediate phenotype, suggesting that the regulatory effects exerted by *Eae23a* and *Eae23b* may be influenced by other regions in the genome. EAE is a multifactorial disease and several regulatory regions and epistatic interactions that influence EAE have previously been demonstrated in the AIL [27], [28]. The AIL contains a genetic heterogeneous background which allows *Eae23* to interact with and be influenced by other loci. Conversely, these effects were fixed in the congenic strain in order to study *Eae23* in isolation. The AIL is therefore likely to capture more accurate genetic effects in the complex setting and thus enable QTL refinement, while the congenic strain represents the isolated effect of alleles at a single locus and thus provides a good tool for dissecting the molecular mechanisms involved in disease.

Out of the 140 genes in *Eae23a*, butyrophilin subfamily 1 member A1 (Btn1a1) is of particular interest because of its effects in EAE. The extracellular Ig-like domain of butyrophilin shares features with MOG that are suggested to allow molecular mimicry, and butyrophilin can induce or suppress the encephalitogenic T cell response to MOG in DA rats [34]. Additionally, butyrophilin can both prevent and suppress MOG-EAE in mice, with subsequent suppression of IFN-γ, IL-2 and IL-12 [35]. The involvement of butyrophilin and other possible candidate genes within *Eae23a* and their effect on EAE requires further investigation that will be pursued in future studies.

For *Eae23b*, we defined *ZEB1* as a possible candidate gene by using isoform-specific expression profiling of the parental DA and PVG.1AV1 and congenic strains. *ZEB1* is a zinc finger homeodomain transcription factor that binds E-box-like sequences at target genes [36] and represses gene transcription by recruiting the C-terminal-binding protein (CtBP) co-repressor [37]–[39]. *ZEB1* is foremost known as an interleukin 2 (*IL2*) repressor [40], [41]. *Zfhep1*, the full-length and fully functional splice-variant of *ZEB1*, can down-regulate expression of its' own gene in addition to other genes. Conversely, splice-variant *Zfhep2* antagonizes this auto-repression by competing for the binding sites without repressing *ZEB1* expression [42]. Thus, increased expression of *Zfhep2* disturbs auto regulation of *Zfhep1* leading to subsequent up-regulation of *Zfhep1* that down-regulates *IL2*. In line with this, we detected higher expression of *Zfhep2* in the lymph node cells of protected DA.PVG-*Eae23* congenic rats 7 days after immunization, when the immune response is mounted against autoantigen. This up-regulation of *Zfhep2* in early disease likely led to disrupted *Zfhep1* auto-regulation in later disease. Consequently, higher levels of *Zfhep1* and lower levels of *IL2* were seen in the protected DA.PVG-*Eae23* rats 35 days after immunization. However, repression of *IL2* expression did not reflect *ZEB1* levels, but depended on *Zfhep1* expression. This finding probably reflects that the full-length variant contains both zinc-finger domains needed to be fully functional [43]. We therefore postulate that the mechanism by which *Eae23b* regulates EAE is through regulation of alternative splicing of *ZEB1* which in turn regulates *IL2*.

*IL2* is involved in T cell development at multiple stages [24], [25], [44]–[46]. The alpha-chain of the receptor for *IL2 (IL2RA)* is a risk gene for MS and other autoimmune diseases [11], [13], [47], [48]. *IL2* was therefore our prime candidate mechanism for *ZEB1* influence on neuroinflammation, supported by finding in the congenic strain. We found decreased levels of Foxp3^+^ cells, which are likely to be Tregs [49], [50], and higher levels of CD8^+^ macrophages in the CNS of disease susceptible DA rats compared to protected congenic rats. This difference was present in rats with the same onset of disease and maximum clinical severity, likely reflecting that DA.PVG-*Eae23* rats recovered from the first bout of disease to a larger extent than DA rats and had milder disease in the chronic phase of EAE (Figure 3). The improved recovery rate observed in congenic rats may reflect the enhanced Treg population. In early disease, we observed a higher potential to expand the Treg population in response to rMOG restimulation in lymph node cells. Consistent with this, a higher number of Tregs were present in the target organ later in disease. Previous studies have reported EAE-suppressive effects of Tregs in the CNS [51], [52]. The higher infiltration rate of Foxp3^+^ cells in protected rats supports the protective role of Tregs and indicates that down-regulated autoimmunity diminished the “inviting signal” to recruit peripheral immune cells that cause disease exacerbation. Accordingly, susceptible rats had higher recruitment of macrophages in the CNS and developed more severe inflammation compared to protected rats. This is possibly a consequence of dysregulation of Tregs in DA rats. This speculation is consistent with involvement of *IL2*. Mice that are deficient of *IL2* and *IL2RA* (*CD25)* mount strong immune responses that contribute to development of autoimmune disease [53], and reduced *IL2* levels correlate with reduced CD4^+^CD25^+^ T cell function [54]. Additionally supporting the involvement of Tregs is that their frequency is normal in MS patients compared to controls, but their suppressive function is diminished [55]. Further study is required to understand the cell populations in the CNS that contribute to disease development and recovery. Therefore, detailed characterization of the kinetics of CNS inflammation in our congenic strain over the course of EAE need to be addressed in future studies. Alternative splicing is a process by which multiple proteins are created from a single pre-mRNA, including exon extensions and deletions [56]–[58]. Bioinformatic analyses suggest that alternative splicing is a significant component in generating diversity of function and predict that the majority of human genes undergo alternative splicing [57]. Splice variants may serve as homeostatic regulators in healthy physiology and disease [59]–[61]. Isoform selection of alternative splice forms may be involved in the pathogenesis of many human diseases and animal models thereof, including cancer and autoimmune diseases [62]–[65]. Cytotoxic T lymphocyte-associated antigen 4 (CTLA4, ENSG00000163599) has demonstrated genetic linkage to a number of autoimmune diseases, including MS [66]–[70], and involves genetic regulation of alternative splice forms [71]. Other genes that regulate disease by differential splice-variant expression are *IL2RA* and *IL7R,* which may involve dysregulation of their membrane-bound and soluble forms in MS patients [12], [72], [73]. For example, the C allele of a risk SNP may contribute to skipping of alternatively spliced exon 6 of *IL7RA* and alter the balance between splice forms [12].

Taken together, these findings indicate that expression and signaling delivered by specific splice variants may play a critical role in regulating autoimmune disease and illustrates the importance of carefully dissecting gene expression data. Concordingly, a SNP in *ZEB1* has recently been suggested to associate with rheumatoid arthritis, another autoimmune disease [74]. We demonstrated that the balance between *Zfhep1* and *Zfhep2*, the splice variants of *ZEB1*, was altered in EAE-susceptible rats when compared to protected rats. Considering the similarities between MOG-EAE and MS, identifying underlying genes may either reveal genes that regulate MS or identify pathogenic pathways of importance for MS. *ZEB1* is a strong candidate because of its regulation of the *IL2* pathway, which is important in MS and EAE. Further functional studies of *ZEB1* and the splice variants may unravel novel pathways contributing to MS pathogenesis.

## Materials and Methods

### Animals

#### Ethics Statement

All experiments in this study were approved and performed in accordance with the guidelines from the Swedish National Board for Laboratory Animals and the European Community Council Directive (86/609/EEC) under the ethical permit N332/06 entitled ‘Genetic regulation, pathogenesis and therapy of EAE, an animal model for multiple sclerosis’, which was approved by the North Stockholm Animal Ethics Committee (Stockholms Norra djurförsöksetiska nämnd). Rats were tested according to a health-monitoring program at the National Veterinary Institute (Statens Veterinärmedicinska Anstalt, SVA) in Uppsala, Sweden. Inbred DA and PVG.1AV1 rats were originally obtained from the Zentralinstitut für Versuchstierzucht (Hannover, Germany) from which colonies have been established at Karolinska Institutet (DA/Kini and PVG.1AV1/Kini). All animals were bred and kept in 12 h light/dark- and temperature-regulated rooms. Rats were housed in polystyrene cages containing aspen wood shavings and had free access to standard rodent chow and water.

#### Advanced Intercross Line

The AIL was established between DA and MHC-identical PVG.1AV1 strains as described previously [28]. Three G10 litters were produced of which 794 rats were selected for MOG-EAE experiments. The litters were similar in size and sex distribution.

#### Congenic strains

The congenic strains were established from DA and PVG.1AV1 rats using a speed-congenic approach with marker-assisted selection [75]. In brief, DA females were mated with male offspring selected for PVG alleles within *Eae23* and against PVG background contamination at 96 microsatellite markers equally spaced throughout the genome (20 centiMorgan intervals). DA females were used throughout the breeding program to ensure that mitochondrial DNA was inherited from DA. A breeding pair selected from the N8 generation was intercrossed to produce homozygous DA.PVG-*Eae23* congenic rats, carrying PVG alleles between D17Rat8 at 27.6 Mb and D17Rat37 at 75.8 Mb (Figure 3).

### Induction and Clinical Evaluation of EAE

Recombinant rat MOG (amino acids 1–125 from the N terminus) was expressed in *Escherichia Coli* and purified to homogeneity by chelate chromatography [76]. Rats between 8 and 11 weeks of age were anesthetized with isoflurane (Abbott Laboratories, Abbott Park, IL, USA) and immunized intradermally in the dorsal tail base. Each animal received a 200 µl inoculum containing MOG in phosphate buffered-saline (PBS) (Life Technologies, Paisley, UK) emulsified 1∶1 with incomplete Freunds adjuvant (IFA) (Sigma-Aldrich, St. Louis, MO, USA). Each MOG batch had slightly different potency, so titration experiments were performed to determine comparable effective doses. AIL rats were immunized with 20 µg of MOG per rat while congenic rats were immunized with sex-specific MOG doses, 15 and 22 µg/rat in females (depending on MOG batch) and 30 µg/rat in males. Animals were weighed and clinical signs of EAE were evaluated daily from day 9–35 p.i. as previously described [28]. Phenotypes used for analysis were incidence of EAE (INC), onset of clinical signs (ONS), duration of symptoms (DUR), maximum and cumulative EAE scores (MAX and CUM, respectively). Weight loss (WL) was calculated by subtracting the lowest weight during the experiment from the weight at time of immunization and expressing the difference as a percentage of the initial weight.

### Genotype and Statistical Analysis

#### Genotyping

Genomic DNA was extracted from tail tips according to a standard protocol [77]. Genotypes were determined by polymerase chain reaction (PCR) amplification of microsatellite markers as previously described [78]. For the AIL, forward primers were end labelled with [γ-^33^P]ATP and the PCR products were size fractionated on 6% polyacrylamide gels and visualized by autoradiography. Primers were obtained from Proligo (Paris, France). For the construction of the congenic strain, fluorophore-conjugated primers were used (Applied Biosystems, Foster City, CA, USA) and PCR products were size fractionated using an electrophoresis capillary sequencer (ABI3730). Genotypes were analyzed using the GeneMapper software (v. 3.7, Applied Biosystems) [79] and all genotypes were manually evaluated by 2 independent observers.

#### Linkage Analysis of AIL

The genetic map was defined using publicly available genome sequence (http://www.ensembl.org/ v.55). Determination of the QTL map position and the confidence interval depends on the correct genomic positions of microsatellite markers. The physical map was used for linkage analysis which enabled comparison of linkage maps of sub-populations. Linkage analysis was performed using R/qtl software version 2.4.1 [80] to identify the QTLs of main effect and two-dimensional scans were performed to identify epistatic interactions. For calculation of linkage to phenotypes the binary model was used for INC and Hailey-Knott regression was used for DUR, MAX, CUM and WL, to generate maximum base 10 logarithm of the likelihood ratio (LOD) scores. Hailey-Knott regression was chosen because it could include covariates, and we tested that the model yielded similar results as a non-parametric or two-part model.

To set the significance threshold, we computed the mean for each family and the residual value from the family mean for each rat and repeated the linkage analysis in the residual data set (indicated in parenthesis in applicable tables) [28]. In addition, the maximum LOD scores for the original one-dimensional scans were all higher than 3.4, a suggested cut-off from simulated data sets [30]. The conventional permutation method [81] could not be used to set the significance thresholds due to the difference in family structure of the G10 compared with F2 generations for which it was developed, while the traditional bootstrapping method [82], [83] could not be used to determine confidence intervals because of the closely situated QTL. Confidence intervals were defined using the closest marker outside a 1.5 LOD support interval from the QTL peak location [84], approximately equivalent to a 95% confidence interval [82].

#### Statistical Analysis

Kruskal–Wallis ranking test was used to confirm the influence of peak markers defined in the linkage analysis on phenotypes, and p values ≤ 0.05 after Bonferroni correction [85] were considered significant. The fit multiple QTL model in R/qtl was used to statistically validate the independent effect of each identified QTL. The probability for each QTL was generated by simulating 1000 pedigrees sampled with replacement from the observed individuals (N = 428), and each was mapped using a single-QTL model with Hailey-Knott regression in R/qtl. Probability was calculated as the percentage of times the maximum LOD (above threshold) was located within the confidence interval of each QTL, as previously described [28]. To analyze the experiments with congenic strains, DA.PVG-*Eae23* was compared with parental DA. To estimate the influence of allele combinations of *Eae23a* and *Eae23b* on disease characteristics, rats from the AIL cohort that carried the genotypes captured in each congenic strain (DA.PVG-*Eae23*, DA.PVG-*Eae23*-Heterozygous and DA) were selected. The expected phenotype values for each congenic strain were based on the average phenotype values within the corresponding AIL group. AIL rats with DA genotype were compared to rats with either PVG or heterozygous genotype in *Eae23*. ONS, MAX, DUR and CUM were analyzed using the Mann–Whitney U-test and Fisher's exact test was used to compare INC. Calculations were carried out using the PC versions of JMP v. 6.0.2 (SAS Institute Inc., Cary, NC, USA). For in vitro experiments data were analyzed using Mann-Whitney's non-parametric ranking test (GraphPad Prism 5.01, GraphPad, San Diego, USA). Median was plotted. Statistical significance was determined as p-value ≤0.05.

### Immunohistochemistry

At day 35 p.i. thirteen DA.PVG-*Eea23* rats and nine DA controls were euthanized by asphyxiation using CO_2_ and perfused with PBS followed by 4% paraformaldehyde. Immunohistochemical analysis of dissected brains and spinal cords was performed on paraformaldehyde fixed paraffin embedded sections. 0.4 mm thick sections were stained with hematoxylin-eosin and luxol fast blue to detect CNS inflammation and demyelination, respectively. The inflammatory index was determined from the number and size of inflammatory lesions on an average of twenty complete cross-sections of the spinal cord and the brain of each animal, as previously described [16].

Staining was performed with antibodies against the following targets: mouse monoclonal α-CD8 (1∶200, Serotec, UK), rabbit monoclonal α-CD3 (1∶150, Thermo Scientific, UK) and rat monoclonal Foxp3 (1∶1000, eBioscience, UK). Incubation with specific primary antibodies was performed overnight at 4°C. Incubation with biotinylated secondary antibody lasted for 1 hour at RT followed by incubation with avidin-horseradish peroxidase complex (HRP; Sigma Aldrich, Germany) for 1 hour at RT. The visualisation of bound biotinylated secondary antibodies was performed with 3,3′ diaminobencidine (DAB)/H_2_O_2_-solution.

### Expression analysis

#### Affymetrix

Four DA and four PVG.1AV1 rats were euthanized using CO_2_ 7 days post-EAE induction, before debut of clinical disease signs. Draining inguinal lymph nodes were collected and samples were prepared as previously described [33]. We used Affymetrix Exon 1.0 ST arrays to assess genome-wide expression. Target labeling, as well as array hybridization, washing and staining were performed as described in the GeneChip Whole Transcript (WT) Sense Target Labeling manual (http://www.affymetrix.com). Arrays were scanned using the GeneChip Scanner 3000 7G (Affymetrix). Probe sequences for all annotated genes within *Eae23* are found in Table S2.

The microarray data is available in MIAME-compliant (minimum information about a microarray experiment) format at the ArrayExpress database (http://www.ebi.ac.uk/arrayexpress) [86] under accession code E-MEXP-2237. CEL intensity files were produced using GeneChip Operating Software version 1.4 (Affymetrix) and quality tested using the Affymetrix Expression Console. Probe-level data were normalized using Robust Multi-array Average (RMA) [87]. Detection of differential expression at the exon and gene level was performed in Partek Genomics Suite 6.4 (Partek Inc., St.Louis, MO, USA). Data was summarized at the gene level using a One-Step Tukey's Biweight Algorithm, which reduces the effect of outlier probe-sets (Statistical Algorithms Description Document; Affymetrix-White-Paper). An ANOVA model, using strain, condition and batch as co-factors, was used to generate raw p values, while FDR was used for multiple test corrections [88]. Genes with 5% FDR were classified as differentially expressed.

#### RNA extraction and RT-PCR

For expression analysis in the parental DA and PVG.1AV1 strains, six females of each strain were immunized with MOG as described above at 12 weeks of age. On day 7 p.i. animals were euthanized using CO_2_ and inguinal lymph nodes were dissected out and snap-frozen in micro tubes and stored at −70°C. For congenic strains, 5 DA and 5 DA.PVG-*Eae23* female rats were sacrificed using CO_2_ at day 7 p.i. and draining inguinal lymph nodes were collected. Lymph nodes were placed in DMEM (Gibco-BRL, Grand Island, NY) enriched with 5% fetal calf serum, 1% L-glutamine, 1% penicillin-streptomycin, 1% pyruvic acid (Life Technologies, Paisley, Scotland) and 50 µM 2-Mercaptoethanol (complete media; Gibco-BRL) before being mechanically separated by passing through a mesh screen with the bolus of a syringe. Cells were spun at 300 g and resuspended in complete media for counting. Cells were allocated to ex vivo flow cytometry, cell culture or RT-PCR. An additional 5 DA and 6 DA.PVG-*Eae23* female rats were sacrificed using CO_2_ at day 7 p.i. and draining inguinal lymph nodes were collected and snap-frozen in micro tubes and stored at −70°C. Furthermore, 10 DA and 13 DA.PVG-*Eae23* females were immunized at 10–12 weeks of age and MOG-EAE was evaluated for 35 days. On day 35 p.i. the rats were euthanized and spleen was dissected out and snap-frozen in micro tubes and stored at −70°C. Tissues were disrupted using Lysing Matrix D tubes (MP Biomedicals, Irvine, CA, USA) on a FastPrep homogenizer (MP Biomedicals).mRNA from cells and homogenates was extracted using RNeasy mini columns (Qiagen Gmbh, Hilden, Germany), including on column DNA-digestion. Reverse transcription was performed using random hexamer primers (Gibco) and Superscript Reverse Transcriptase (Invitrogen, Carlsbad, CA, USA). Real-time PCR was performed using an ABI7900HT Fast Real-Time PCR System (Applied Biosystems Inc., Foster City, CA, USA) with a two-step PCR protocol (50°C for 2 min and 95°C for 10 min followed by 40 cycles of 95°C for 15 sec and 60°C for 60 sec), using SYBR green as reporter dye (Applied Biosystems Inc.). Serial 2-fold dilutions from a pool of ConA stimulated DA lymphocyte cDNA was used as standard for assessment of relative efficiency of target amplification. We used the ΔΔCT method for relative quantification as the PCR efficiencies between the target(s) and reference gene(s) were relatively equivalent. Relative quantity (RQ) of expression was calculated as 2 ^−ΔΔCt^ (ΔΔCt  =  (target gene - reference gene) – average target gene). Primers sequences for the quantitative real time PCR are found in Table S3.

### *In vitro* experiment

#### Stimulation

For stimulation experiments 1×10^6^ cells/well were plated in 48 well flat bottom plates (Nunc, Roskilde, Denmark). Cells were stimulated with either: complete media, 0.5 µg/ml Concanavalin A (Sigma-Aldrich) or 25 µg/ml rMOG for 24 hrs at 37°C and 5% CO_2_. Supernatants were collected for IL-2 ELISA. Cells were spun down and used for flow cytometry.

#### IL-2 Antibody Enzyme Linked ImmunoSorbent Assays

IL-2 ELISAs were performed according to manufacturers' recommendations (Biosource International, California, USA) using 96-well flat-bottom ELISA plates (Nunc). Supernatants from stimulation assays were collected at 24 hrs.

#### Flow Cytometry

Lymph node cells were washed with cold PBS and resuspended in a further 100 µl of PBS. Cells were first stained for extracellular CD4-PE-Cy5 and CD25-PE (20 min at 4°C; BD Biosciences, San Jose, USA) and thereafter fixed and stained for intracellular Foxp3-APC according to manufacturers' recommendations (eBioscience, San Diego, USA). Staining was visualized on a FACSort (BD, Franklin Lakes, USA) with Cell Quest (version 3.2.1f1, BD) and analyzed using FloJo (version 8.8).

## Supporting Information

Figure S1 Summary of phenotype distribution in (DAxPVG.1AV1)G10 AIL rats. The y-axis shows the number of rats with each phenotypic value. The clinical parameters are detailed in Materials and Methods. A-B: Distribution of all 794 (DAxPVG.1AV1)G10 rats. C-G: Distribution of 224 (DAxPVG.1AV1)G10 rats that developed EAE. Abbreviations: M  =  males, F  =  females, INC  =  incidence of EAE, WL  =  weight loss, CUM  =  cumulative EAE score, MAX  =  maximum EAE score, DUR  =  duration of EAE, ONS  =  day of onset of EAE, p.i.  =  post immunization.
(0.14 MB TIF)

Figure S2 Eae23 regulates EAE in AIL rats although males have low power. Log-likelihood plots of Eae23 in 794 (DAxPVG.1AV1)G10 AIL rats. Linkage analysis performed in groups stratified according to sex identified two separate QTLs strongly linked to EAE phenotypes in females while males showed one centered QTL below the threshold for significance. To investigate the possibility of sex-specific QTLs or sex interacting with genotype, we included sex as a covariate in the model. Sex was not significantly contributing to the linkage of Eae23 to clinical phenotypes. Males weighed more than females at the age of immunization and the dose used was possibly suboptimal to induce EAE in the male population, which reduced the power to separate the QTLs in the joint analysis. Microsatellite marker positions are indicated by vertical lines on the x-axis. A) Phenotype codes: INC  =  light grey; ONS  =  dark grey; MAX  =  black; DUR  =  black dashed; CUM  =  grey. B) Data stratified according to sex compared with the complete cohort. MAX is a representative for all clinical phenotypes. Group codes: All (N  =  794)  =  black; Females (N  =  428)  =  red; Males (N  =  366)  =  blue. C) Linkage analysis including sex as a covariate. Hailey-Knott regression model  =  black; Hailey-Knott regression model including sex as an additive covariate  =  blue dashed line; Hailey-Knott regression model including sex as an interactive covariate  =  red dotted line. MAX is a representative for all clinical phenotypes. Marker location/information was retrieved from Ensembl Genome Database (http://www.ensembl.org v.55).
(0.15 MB PDF)

Figure S3 IL-2 protein expression in DA.PVG-Eae23 and parental DA. IL-2 protein expression in lymph nodes collected day 7 p.i., measured by Antibody Enzyme Linked ImmunoSorbent Assays (ELISA), reveals no differences between DA.PVG-Eae23 and parental DA. Median values are indicated by horizontal bars. Mann-Whitney U-test was used to compare IL-2 levels; p values ≤0.05 were considered significant. Abbreviations: D.P-Eae23  =  DA.PVG-Eae23, ConA  =  Concanavalin A, rMOG  =  recombinant myelin oligodendrocyte glycoprotein. A) IL-2 protein concentration in picograms per milliliter in supernatant from ConA stimulated lymph node cells. B) IL-2 protein concentration in picograms per milliliter in supernatant from rMOG stimulated lymph node cells.
(0.70 MB TIF)

Table S1 Primer sequences were retrieved from Ensembl Genome Database (http://www.ensembl.org v.55). a Positions are given in megabasepair. The skewing of genotype distributions reflect regions of genome that are becoming fixed in the population due to the genetic drift within the breeding couples used (50 per generation). The region between markers D17Got49 and D17Mgh5 has an overrepresentation of DA/DA genotype (∼29% in the 7th AIL generation and ∼35% in the 10th AIL generation) and marker D17Rat95 has drifted toward PVG/PVG genotype. The minimum number of individuals with a particular genotype was 68 rats, which should be sufficient for linkage. Additionally, genotyping of selected markers within these regions were repeated to ensure that genotypes were correctly scored.
(0.04 MB DOC)

Table S2 mRNA expression in lymph nodes collected day 7 p.i. from parental DA and PVG.1AV1 measured by Affymetrix Exon 1.0 ST arrays. Data was summarized at the gene level using a One-Step Tukey's Biweight Algorithm and an ANOVA model was used to generate raw p values. Fold change was calculated as DA expression/PVG.1AV1 expression.
(0.04 MB DOC)

Table S3 (0.03 MB DOC)
