# Supplementary figures and images for: Fine-Mapping Resolves Eae23 into Two QTLs and Implicates ZEB1 as a Candidate Gene Regulating Experimental Neuroinflammation in Rat
Source: PLoS One. 2010 Sep 15;5(9):e12716. doi: 10.1371/journal.pone.0012716 (PMC2939884; doi:10.1371/journal.pone.0012716)

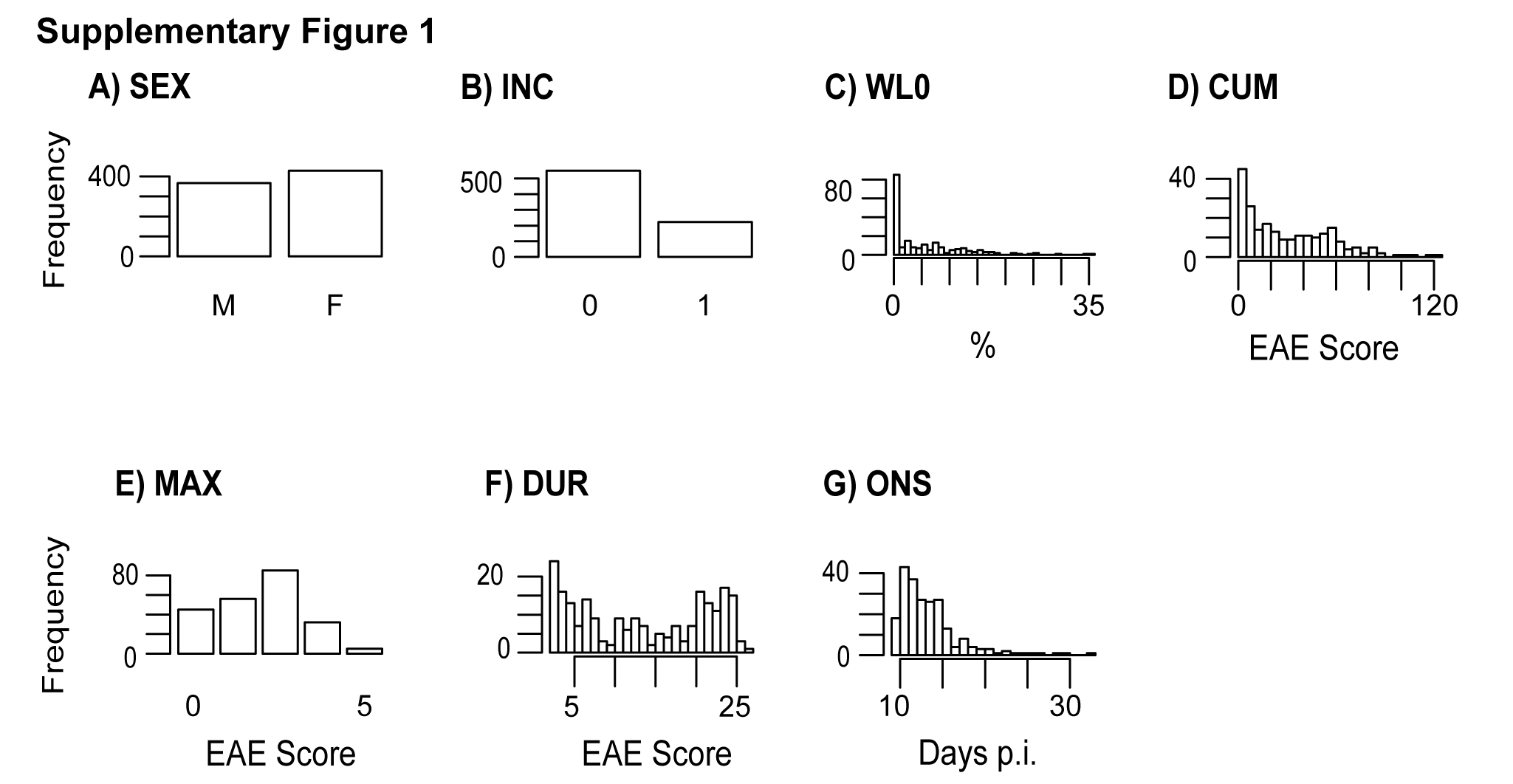

Supplement: Figure S1 — Summary of phenotype distribution in (DAxPVG.1AV1)G10 AIL rats. The y-axis shows the number of rats with each phenotypic value. The clinical parameters are detailed in Materials and Methods. A-B: Distribution of all 794 (DAxPVG.1AV1)G10 rats. C-G: Distribution of 224 (DAxPVG.1AV1)G10 rats that developed EAE. Abbreviations: M = males, F = females, INC = incidence of EAE, WL = weight loss, CUM = cumulative EAE score, MAX = maximum EAE score, DUR = duration of EAE, ONS = day of onset of EAE, p.i. = post immunization. (0.14 MB TIF) [file pone.0012716.s001.tif]

Supplementary Figure 2

A)

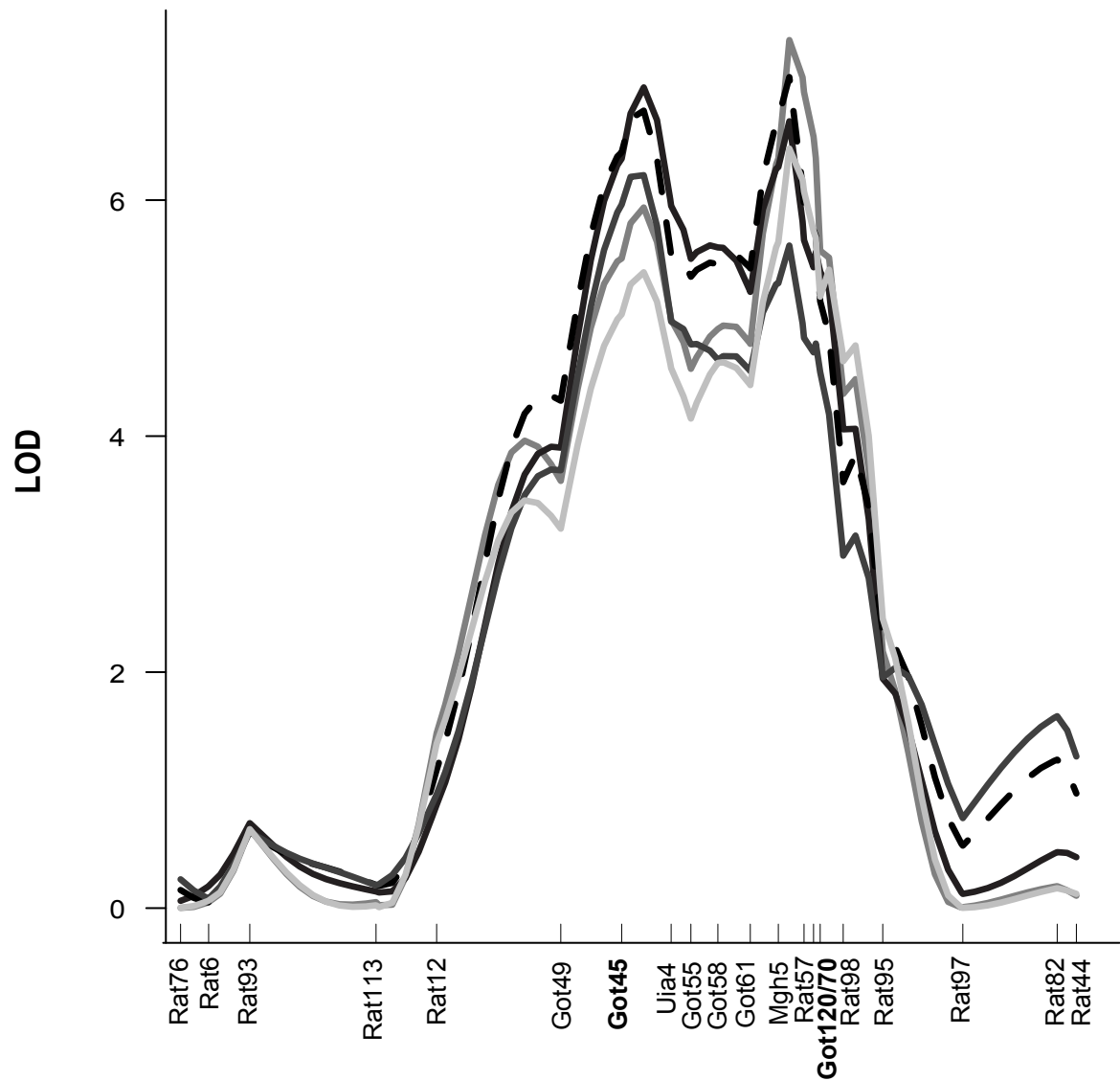

B)

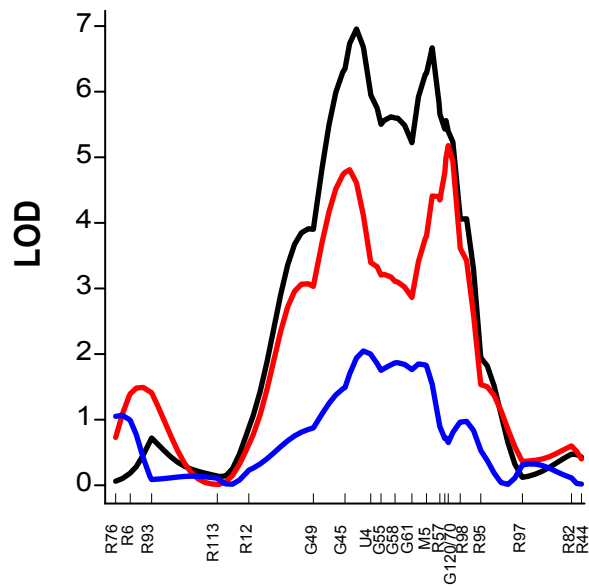

C)

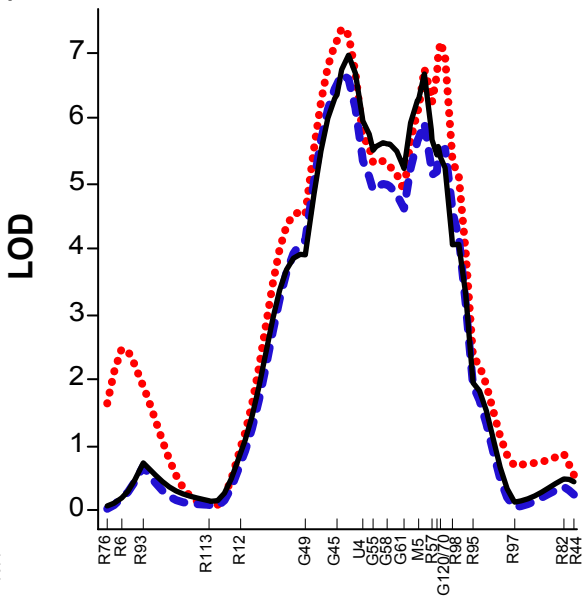

Supplement: Figure S2 — Eae23 regulates EAE in AIL rats although males have low power. Log-likelihood plots of Eae23 in 794 (DAxPVG.1AV1)G10 AIL rats. Linkage analysis performed in groups stratified according to sex identified two separate QTLs strongly linked to EAE phenotypes in females while males showed one centered QTL below the threshold for significance. To investigate the possibility of sex-specific QTLs or sex interacting with genotype, we included sex as a covariate in the model. Sex was not significantly contributing to the linkage of Eae23 to clinical phenotypes. Males weighed more than females at the age of immunization and the dose used was possibly suboptimal to induce EAE in the male population, which reduced the power to separate the QTLs in the joint analysis. Microsatellite marker positions are indicated by vertical lines on the x-axis. A) Phenotype codes: INC = light grey; ONS = dark grey; MAX = black; DUR = black dashed; CUM = grey. B) Data stratified according to sex compared with the complete cohort. MAX is a representative for all clinical phenotypes. Group codes: All (N = 794) = black; Females (N = 428) = red; Males (N = 366) = blue. C) Linkage analysis including sex as a covariate. Hailey-Knott regression model = black; Hailey-Knott regression model including sex as an additive covariate = blue dashed line; Hailey-Knott regression model including sex as an interactive covariate = red dotted line. MAX is a representative for all clinical phenotypes. Marker location/information was retrieved from Ensembl Genome Database (http://www.ensembl.org v.55). (0.15 MB PDF) [file pone.0012716.s002.pdf]

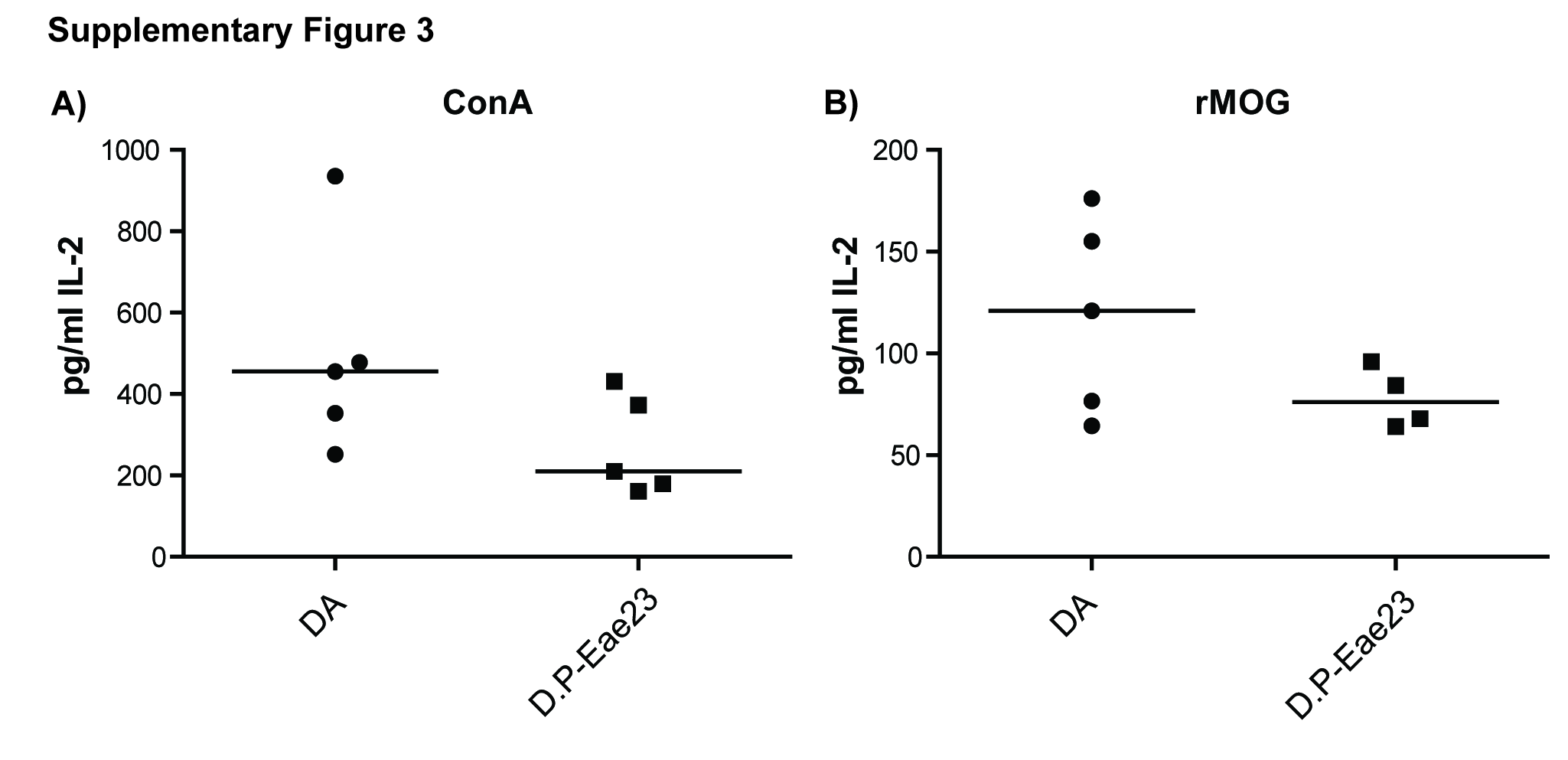

Supplement: Figure S3 — IL-2 protein expression in DA.PVG-Eae23 and parental DA. IL-2 protein expression in lymph nodes collected day 7 p.i., measured by Antibody Enzyme Linked ImmunoSorbent Assays (ELISA), reveals no differences between DA.PVG-Eae23 and parental DA. Median values are indicated by horizontal bars. Mann-Whitney U-test was used to compare IL-2 levels; p values ≤0.05 were considered significant. Abbreviations: D.P-Eae23 = DA.PVG-Eae23, ConA = Concanavalin A, rMOG = recombinant myelin oligodendrocyte glycoprotein. A) IL-2 protein concentration in picograms per milliliter in supernatant from ConA stimulated lymph node cells. B) IL-2 protein concentration in picograms per milliliter in supernatant from rMOG stimulated lymph node cells. (0.70 MB TIF) [file pone.0012716.s003.tif]
