# Supplementary material for: Fine-Mapping Resolves Eae23 into Two QTLs and Implicates ZEB1 as a Candidate Gene Regulating Experimental Neuroinflammation in Rat
Source: PLoS One. 2010 Sep 15;5(9):e12716. doi: 10.1371/journal.pone.0012716 (PMC2939884; doi:10.1371/journal.pone.0012716)
Supplement: Table S1 — Primer sequences were retrieved from Ensembl Genome Database (http://www.ensembl.org v.55). a Positions are given in megabasepair. The skewing of genotype distributions reflect regions of genome that are becoming fixed in the population due to the genetic drift within the breeding couples used (50 per generation). The region between markers D17Got49 and D17Mgh5 has an overrepresentation of DA/DA genotype (∼29% in the 7th AIL generation and ∼35% in the 10th AIL generation) and marker D17Rat95 has drifted toward PVG/PVG genotype. The minimum number of individuals with a particular genotype was 68 rats, which should be sufficient for linkage. Additionally, genotyping of selected markers within these regions were repeated to ensure that genotypes were correctly scored. (0.04 MB DOC) [file pone.0012716.s004.doc]

Supplementary Table 1. Microsatellite markers used for genotyping.

| Marker | Position^a^ | % Genotyped | | | |
| --- | --- | --- | --- | --- | --- |
|  |  | Total | DA/DA | DA/PVG | PVG/PVG |
| D17Rat76 | 13.930241 | 93 | 25 | 52 | 22 |
| D17Rat6 | 16.053310 | 93 | 27 | 51 | 22 |
| D17Rat93 | 19.162955 | 90 | 27 | 50 | 22 |
| D17Rat113 | 28.674573 | 91 | 28 | 50 | 21 |
| D17Rat12 | 33.277332 | 94 | 24 | 54 | 21 |
| D17Got49 | 42.659521 | 92 | 34 | 46 | 20 |
| D17Got45 | 47.261860 | 91 | 40 | 43 | 17 |
| D17Uia4 | 51.224822 | 91 | 33 | 50 | 17 |
| D17Got55 | 52.482956 | 87 | 34 | 49 | 17 |
| D17Got58 | 54.533049 | 87 | 34 | 50 | 16 |
| D17Got61 | 56.974438 | 92 | 33 | 49 | 18 |
| D17Mgh5 | 59.104848 | 92 | 34 | 45 | 21 |
| D17Rat57 | 61.030026 | 97 | 26 | 49 | 24 |
| D17Got120 | 61.762698 | 92 | 25 | 51 | 23 |
| D17Got70 | 62.262606 | 93 | 23 | 54 | 22 |
| D17Rat98 | 64.047700 | 94 | 26 | 51 | 23 |
| D17Rat95 | 66.993031 | 88 | 16 | 52 | 32 |
| D17Rat97 | 73.041112 | 93 | 24 | 47 | 29 |
| D17Rat82 | 80.162496 | 96 | 30 | 50 | 20 |
| D17Rat44 | 81.612362 | 96 | 24 | 51 | 25 |
