# Supplementary material for: Fine-Mapping Resolves Eae23 into Two QTLs and Implicates ZEB1 as a Candidate Gene Regulating Experimental Neuroinflammation in Rat
Source: PLoS One. 2010 Sep 15;5(9):e12716. doi: 10.1371/journal.pone.0012716 (PMC2939884; doi:10.1371/journal.pone.0012716)
Supplement: Table S2 — mRNA expression in lymph nodes collected day 7 p.i. from parental DA and PVG.1AV1 measured by Affymetrix Exon 1.0 ST arrays. Data was summarized at the gene level using a One-Step Tukey's Biweight Algorithm and an ANOVA model was used to generate raw p values. Fold change was calculated as DA expression/PVG.1AV1 expression. (0.04 MB DOC) [file pone.0012716.s005.doc]

**Supplementary Table 2. Expression of *Eae23*** genes.

| **Gene** | **P value** | **Expression Fold-Change** |
| --- | --- | --- |
| Arid4b (**ENSRNOG00000016391)** | 0.13 | 1.30 |
| Ggps1 (**ENSRNOG00000016767)** | 0.28 | 1.29 |
| Tbce (**ENSRNOG00000029667)** | 0.14 | 1.29 |
| B3galnt2 (**ENSRNOG00000016855)** | 0.92 | 1.01 |
| Kif5b (**ENSRNOG00000017466)** | 0.71 | -1.04 |
| NP_001100827.1 (**ENSRNOG00000017791)** | 0.86 | -1.03 |
| Zeb1 (**ENSRNOG00000017863**) | 0.74 | 1.09 |
| ENSRNOG00000038651 | 0.70 | 1.08 |
| NP_001100828.1 **(ENSRNOG00000028329)** | 0.25 | -1.28 |
| NP_001101886.1 **(ENSRNOG00000018110)** | 0.29 | -1.07 |
| NP_001100829.1 **(ENSRNOG00000016578)** | 0.67 | -1.12 |
| Map3k8 **(ENSRNOG00000016378)** | 0.91 | 1.01 |
| ENSRNOG00000030744 | 0.08 | -1.12 |
| Bambi **(ENSRNOG00000016066)** | 0.29 | 1.26 |
| RGD1564129_predicted **(ENSRNOG00000027453)** | 0.74 | -1.05 |
| NP_001101887.1 **(ENSRNOG00000015292)** | 0.70 | -1.05 |
| Crem **(ENSRNOG00000014900)** | 0.50 | 1.10 |
| Epc1 **(ENSRNOG00000014834)** | 0.39 | -1.40 |
| ENSRNOG00000029573 | 0.68 | 1.11 |
| Rab18 **(ENSRNOG00000018972)** | 0.34 | 1.21 |
| Irxl1_predicted **(ENSRNOG00000018938)** | 0.83 | -1.02 |
| Armc4_predicted **(ENSRNOG00000018905)** | 0.41 | 1.13 |
| Mpp7 **(ENSRNOG00000018760)** | 0.37 | -1.14 |
