# Supplementary material for: Fine-Mapping Resolves Eae23 into Two QTLs and Implicates ZEB1 as a Candidate Gene Regulating Experimental Neuroinflammation in Rat
Source: PLoS One. 2010 Sep 15;5(9):e12716. doi: 10.1371/journal.pone.0012716 (PMC2939884; doi:10.1371/journal.pone.0012716)
Supplement: Table S3 — (0.03 MB DOC) [file pone.0012716.s006.doc]

**Supplementary Table 3. Primer sequences for RT-PCR.**

| Gene | Forward Primer | Reverse Primer |
| --- | --- | --- |
| Beta-Actin | 5’CGTGAAAAGATGACCCAGATCA3’ | 5’AGAGGCATACAGGGACAACACA3’ |
| GAPDH | 5’TCAACTACATGGTCTACATGTTCCAG3’ | 5’TCCCATTCTCAGCCTTGACTG3’ |
| HPRT | 5’CTCATGGACTGATTATGGACA3’ | 5’GCAGGTCAGCAAAGAACTTAT3’ |
| IL2 | 5’GCGTGTGTTGGATTTGACTC3’ | 5’ACAGTTGCTGGCTCATCATC3’ |
| ZEB1 | 5’AATATGAGCATACAGGTAAGA3’ | 5’CACACTTGTCACATTGGTAGG3’ |
| Zfhep1 | 5’GTCATGATGACAATGGAACA3’ | 5’TGACGAATGCGACTCAGATG3’ |
| Zfhep 2 | 5’CATCTGAAGGAGCACTTACGG3’ | 5’TACCATTCACAGGCATCAAGC3’ |
